# Population pharmacokinetic analysis for dabigatran etexilate in Chinese patients with non-valvular atrial fibrillation

**DOI:** 10.3389/fcvm.2022.998751

**Published:** 2022-10-28

**Authors:** Ya-ou Liu, Qiu-fen Xie, Zhi-yan Liu, Zhe Wang, Guang-yan Mu, Ya-tong Zhang, Zi-nan Zhao, Dong-dong Yuan, Li-ping Guo, Na Wang, Jing Xiang, Hong-tao Song, Jie Jiang, Qian Xiang, Yi-min Cui

**Affiliations:** ^1^Department of Pharmacy, Peking University First Hospital, Beijing, China; ^2^School of Pharmaceutical Sciences, Peking University Health Science Center, Beijing, China; ^3^Department of Pharmacy, Beijing Hospital, Beijing, China; ^4^Department of Pharmacy, Zhengzhou Seventh People's Hospital, Zhengzhou, China; ^5^Department of Pharmacy, The Second Affiliated Hospital of Chongqing Medical University, Chongqing, China; ^6^Department of Pharmacy, 900 Hospital of the Joint Logistics Team, Fuzhou, China; ^7^Department of Cardiology, Peking University First Hospital, Beijing, China; ^8^Institute of Clinical Pharmacology, Peking University, Beijing, China

**Keywords:** population pharmacokinetics, pharmacodynamics, bleeding, dabigatran, non-valvular atrial fibrillation

## Abstract

We aimed to develop a pharmacokinetic (PK) and pharmacodynamic (PD) model from healthy Chinese subjects and real-world non-valvular atrial fibrillation (NVAF) patients. We also investigated meaningful intrinsic and extrinsic factors and related biomarkers for bleeding events. We characterized the integrated PK/PD models based on rich PK/PD data [dabigatran concentration, activated partial thromboplastin time (APTT), prothrombin time (PT), and anti-factor IIa (anti-FIIa) activity] from 118 healthy volunteers and sparse PD data [APTT, PT, and anti-FIIa] from 167 patients with NVAF after verifying the model extrapolation performance. We also documented the correlations between PD biomarkers and clinically relevant bleeding events over one year. Next, we used the final integrated PK/PD model (a two-compartment, linear model with first-order absorption) to evaluate the influence of dosage and individual covariates on PD parameters. The age, high-density liptein cholesterol (HDL-C), and creatinine clearance (CrCL) improved the PK model fit. The linear direct-effects PD model described the correlation between APTT, PT, and anti-FIIa and plasma concentration. CrCL improved the PD model fit. Anti-FIIa was more sensitive to the increase in dabigatran exposure than APTT and PT in the PD model. Therefore, fixed dabigatran doses could be prescribed for patients with NVAF without adjusting for age and HDL-C. We observed an elevated bleeding tendency with higher peak and trough values of APTT, PT, and anti-FIIa. Randomized studies should be performed to evaluate the efficacy and safety of low-dose dabigatran in Chinese patients with NVAF.

## Introduction

In patients with atrial fibrillation (AF), a thromboembolic event is one of the main factors increasing the long-term disability and mortality risks (1); thus, many national guidelines recommend direct-acting oral anticoagulants (DOACs) as the first-line therapy (1–4). Dabigatran etexilate, the prodrug of dabigatran (Class IB recommendation), directly inhibits thrombin (factor IIa) activity and was approved for the prevention of stroke and systemic embolism in patients with non-valvular AF (NVAF) in 2010 (5). Furthermore, unlike Vitamin K Antagonists, dabigatran can be administered with fixed-dose regimens, requires no regular monitoring, and has few food and drug interactions (6). Considering these advantages, generic drug development, and drug price reductions, dabigatran etexilate, as an important DOAC, has become more widely prescribed in the Chinese healthcare system in the past year (7).

However, whether the fixed-dose regimen of dabigatran etexilate is applicable to all patients with NVAF is still being explored. Although the RE-LY (Randomized Evaluation of Long-term Anticoagulant Therapy) study applied regimens of 110 and 150 mg twice daily (6), the approved drug specifications by the United States of America (USA) Food and Drug Administration (FDA) were 75 and 150 mg per capsule. In comparison, Chinese authorities approved the 110 and 150 mg per capsule formulations. Besides, while, in the USA, no dosage adjustment is necessary, in China and Japan, the lower doses of 110 mg twice daily are recommended for patients with NVAF and creatinine clearance rates (CrCL) of 30–50 mL/min (8). However, the FDA suggests using 75 mg twice daily for patients with NVAF and CrCL of 15–30 mL/min, while China and Japan have no such instructions. Moreover, dabigatran differently affects Asian and non-Asian populations (9). Compared to non-Asian patients, Asian patients with dabigatran 110 or 150 mg twice daily reduced the risk of stroke and systemic embolization, hemorrhagic stroke as well as all-cause mortality, whereas had no increased risk of gastrointestinal bleeding (10).

Thus, the interindividual variations of dabigatran have been demonstrated, and they involve many intrinsic and extrinsic factors (11). In AF patients, the interindividual coefficient of variation in the plasma concentration of dabigatran was 51–64%, while the intraindividual one was 32–40% (12). Meanwhile, in healthy volunteers, the intersubject coefficient of variation was about 30% in pharmacokinetic (PK) and < 10% in pharmacodynamic (PD) parameters (13). Some studies have revealed close correlations between dabigatran plasma concentration and PD parameters, including activated partial thromboplastin time (APTT) and anti-factor IIa (anti-FIIa) activity (5). Considering the high risk of bleeding, the relationship between the clinical outcomes of bleeding and PK or PD indexes for patients with NVAF receiving dabigatran remains unclear. Thus, documenting factors and biomarkers related to dabigatran-induced bleeding is necessary.

For the above purposes, previous studies have focused on population PK (PopPK) analysis in AF patients (14–16). Although these studies involved PK modeling and simulation, they did not include healthy Chinese volunteers. Besides, AF patients were mainly from the premarket studies (such as the RE-LY or PETRO studies) by sponsors (6, 17), not from real-world studies. Moreover, only one study contained the PD index of APTT (15), and none focused on the bleeding events. Therefore, the purpose of our study was first to establish a PopPK model and PK/PD model based on the PK and PD data of healthy Chinese volunteers; second, to verify the extrapolation applicability of the volunteer model based on the sparse PK data of patients; and finally, to establish a comprehensive PK/PD model based on the PD data of volunteers and patients. In addition, we discussed the correlation between the comprehensive PK/PD model and cumulative clinical bleeding events and evaluated the influence of selected factors. Finally, in a virtual population of patients with NVAF, we used our model to simulate PD indicators and compared the obtained ranges with the relevant guidelines.

## Methods

### Study design

Studies on healthy Chinese participants (study 1) and patients (study 2) conducted across multiple centers were used for the popPK and PK/PD analysis of dabigatran etexilate (Supplementary Table 1). The ethics committee of Peking University First Hospital and all the participating subcentral hospitals approved the protocol of our study registered in ClinicalTrial.org (NCT03161496). Before the beginning of the research, all subjects were briefed about the study process and provided written informed consent.

The single-center study on healthy volunteers was based on a bioequivalence trial of dabigatran etexilate with a four-period crossover design conducted in China from 2018 to 2019 (5). Eligible healthy volunteers (18–65 years old) meeting all the inclusion and none of the exclusion criteria of the bioequivalence trial received a single dabigatran etexilate capsule (150 mg) either in fasting condition or within 30 min after a standard high-fat meal (5). The venous blood samples (4 mL) in EDTA-K_2_ tubes were collected before administration and then at 17–18 time points for plasma dabigatran quantification. For PD parameters tests, we collected extra blood samples (2.7 mL) in sodium citrate (3.2% v/v) tubes at 0, 2, 8, and 12 h after drug administration in any one period of reference formulation (brand name: Pradaxa^®^). The PK parameters for modeling and simulation were also from the same period.

The multicenter and prospective study was performed in five Chinese hospitals. Inclusion Criteria: Conform to the anticoagulation indication of NOACs; Age is >18, male or female are fine; those who have taken rivalsaban, apixaban or dabigatran continuously for more than 1 week. Exclusion criteria: History of immunodeficiency diseases, including positive HIV test results; Positive results of hepatitis B surface antigen (HBsAg) or hepatitis C; Combination of powerful CYP3A4 inhibitors and P-glycoprotein (P-gp) inhibitors within 14 days before NOACs treatment, Strong CYP3A4 inducer and P-glycoprotein (P-gp) inducer (Supplementary Table 2); Severe liver and kidney dysfunction; Contains contraindications to NOACs anticoagulation. We collected blood samples (2.7 mL) >10 h after the previous dabigatran dose for trough PD tests and 2 h after dabigatran administration for peak PD tests (at steady state and without any dose adjustment). For a few patients, and with their agreement, we also collected 2 mL blood samples in EDTA-K_2_ tubes at the same two time points as above to detect plasma concentrations for model verification. We regularly followed up on the enrolled patients through phone or office appointments for a year. The primary outcome was any clinically relevant bleeding event evaluated by two independent physicians based on the criterion of the Bleeding Academic Research Consortium (BARC) (18).

### Determination of PK and PD parameters

For healthy volunteers, we centrifuged the blood samples for PK parameters detection for 10 mins at 4°C and 1,700 g within 1 h after collection. We then transferred the plasma samples to cryovials and stored them at −80°C. We quantified plasma concentrations of total dabigatran in the subcenter through a validated previously published liquid chromatography-tandem mass spectrometry (LC-MS/MS) method (5).

We hydrolyzed dabigatran acyl-β-d-glucuronic acid to free dabigatran by alkaline heating, and obtained reliable conversion efficiency to support accurate determination of total dabigatran.

For patients, we centrifuged blood samples for PK model verification for 10 mins at room temperature and 3,000 rpm within 1 h after collection. We also quantified plasma concentration of total dabigatran with an LC-MS/MS method in another subcenter. The analytical column was an UItimate XB-C8 HPLC Column (3.0 × 250 mm). Mobile phase A was a 0.1% formic acid aqueous solution, and mobile phase B was 100% acetonitrile with 0.1% formic acid. The injection volume was 1 μL, and the flow rate was 320 μL/min. The column temperature was maintained at 30°C, and that of the sample chamber was 4°C. The linear calibration range was 2–500 ng/ml. The three quality control sample concentrations were 5, 50, and 325 ng/ml, and their precisions (±relative standard deviation) were 4.73 ± 9.93%, 45.23 ± 7.17%, and 433.26 ± 11.74%, respectively. The interbatch and introbatch accuracy of dabigatran ranged from 80.95% to 111.63%, and the absolute recovery was within 83.2–88.6%. We found no significant matrix effect. We analyzed all the samples within established storage stability periods.

For all the subjects, we centrifuged the blood samples for PD parameters determination for 15 min at room temperature and 2,500 g within 1 h after collection. After transferring the plasma samples to cryovials, we stored them at −80°C and transferred them to the Peking University First Hospital for PD analysis within 6 months. We determined the PD parameters, including prothrombin time (PT), APTT, and anti-FIIa activity using an automated multiparameter hemostasis analyzer (Sysmex^®^ CS-2100i) as previously published (19, 20).

### PopPK and PK/PD model development

We developed the PopPK and PK/PD models using the non-linear mixed-effect modeling tool NONMEM (version 7.2.0, icon development solution, Ellicott, Maryland, USA). We performed all model operations using the interaction method (FOCE INTER), with interindividual variation – residual variation (η – ε) for first-order conditional estimation. The model selection was based on the likelihood ratio test, residual analysis, and parameter rationality analysis. If the likelihood ratio test yielded a *p*-value >3.84, we added covariates to the basic model (α ≤ 0.05, when there is one degree of freedom, the change of objective function value is >3.84). In contrast, if the likelihood ratio test yielded a *p*-value lower than 6.63, we removed the covariates from the complete model (α ≤ 0.01, when there is one degree of freedom, the change of objective function value is >6.63). We performed statistical analysis using R (version 4.1.4, R statistical calculation basis), Xpose (version 4.4.0), and PSN (version 3.2.12) (21).

We first developed the PopPK model based on the data from the healthy volunteers and then modeled the APTT, PT, and anti-FIIa activity time curves of dabigatran in healthy volunteers using the Bayesian estimation pharmacokinetic parameters of each individual (22, 23). Using this PK/PD model, we conducted external verification based on patient PK data to confirm the feasibility of extrapolating data from volunteers to patients. Finally, we included the patient PD data in the original model to develop a comprehensive PK/PD model.

### Healthy participants' PopPK model

Based on the literature, we described the PK of dabigatran etexilate using a two-compartment model (14, 15, 24). We excluded concentrations below the lower limit of quantification from the analysis.

We modeled interindividual variability (IIV) as an exponential error term. Assuming that the model parameters obey lognormal distribution, we modeled the IIV of each structural parameter in NONMEM, as follows:


$$\begin{array}{l} \text{Equation }1:\theta_{i}\;=\;\theta_{tv}*\exp^{\eta i} \end{array}$$

where θ_i_ represents the parameter of the i^th^ individual, θ_tv_ is the typical value of the parameter in the group, and η_i_ is random variables with a mean of zero and a variance of ω^2^.

Residual variability is described by additive proportional error, as follows:


$$\begin{array}{l} \text{Equation }2:C_{ij}\;=\;C_{pred,ij}*\left(1+\varepsilon_{1,pred,ij}\right)+\varepsilon_{2,pred,ij} \end{array}$$

where C_ij_ represents the predicted value for the i^th^ individual, C_pred, ij_ are the typical predicted values of the parameter in the population, ε_1, pred, ij_ are proportional prediction errors, and ε_2, pred, ij_ are additive prediction errors.

We performed covariate analysis after selecting the basic model. We used demographic factors [age, sex, body weight, body mass index, etc.], liver function markers [alanine aminotransferase, aspartate aminotransferase, alkaline phosphatase, hemoglobin], renal function markers [serum creatinine, endogenous ClCR, etc.], and blood lipid and diet indicators [triglyceride, total cholesterol, low-density lipoprotein, high-density lipoprotein (HDL-C), diet effect, etc.] as potential covariates of central compartment apparent volume (VC/F) and apparent systemic clearance (CL/F).

Using the model with continuous or classification covariates requires equation 3 or 4, respectively:


$$\begin{array}{l} \text{Equation }3:\theta_{i}\;=\;\theta_{tv}*\exp^{\eta i}*{\theta_{cov}}^{\frac{COV_{i}}{COV_{pop}}}\\ \text{Equation }4:\theta_{i}\;=\;\theta_{tv}*\exp^{\eta i}*IND*\theta_{cov} \end{array}$$

where θ_i_ represents the parameter of the i^th^ individual, θ_tv_ represents the typical value of the parameter, θ_cov_ represents the typical value of the covariate parameters, and η_i_ represents random variables with a mean of zero and a variance of ω^2^. COV_i_ is a continuous covariate, and COV_pop_ is the median of continuous covariates. If there is a classification covariate, IND = 1; if there is no classification covariate, IND = 0.

### Healthy participants' PK/PD modeling

We modeled APTT, PT, and anti-FIIa activity data using a single empirical maximum *a posteriori* probability Bayes PK parameter estimation as input. Based on the literature, the linear direct effect model is suitable to describe the PK/PD data of dabigatran using the fitting equation:


$$\begin{array}{l} \text{Equation }5:PD=E_{0}+K\times X_{1} \end{array}$$

where E0 represents the PD baseline (E0 for anti-FIIa activity fixed 0), K is the slope of the linear intercept model, and X1 is the plasma concentration of dabigatran predicted by the Bayesian method.

We modeled IVV as exponential error terms on APTT, PT, and anti-FIIa activity. For residual variability, APTT, PT, and anti-FIIa activity all used additive error models.

We systematically evaluated the effects of demographic factors, liver function, renal function, blood lipid, and diet through progressive forward selection and backward elimination.

### Integrated PK/PD modeling

Using the data of healthy volunteers and patients, we established a comprehensive PK/PD model. During joint analysis, we did not modify the PK structure model of healthy volunteers. Because the patient PK study only provided sparse data and was only used for model extrapolation verification, we used the volunteer PK model and extrapolated it to patients. The PD study included the PD data of healthy people and patients for model recalculation, and PD recalculation parameters reflected the population differences between volunteers and patients.

### Model evaluation

We used the self-lifting sampling technology of bootstrap to simulate the PK/PD curve 1,000 times and compared the results with the observed data to evaluate the model's prediction performance. Using a standard goodness-of-fit (GoF) diagram, we evaluated the final PK and PK/PD models. We calculated the shrinkage of interindividual and residual variation terms to provide additional information about the fitting characteristics of the model. We evaluated the prediction performance of the model by simulating data based on the final model and performed prediction-corrected visual prediction checks (pcVPC) between the observation data and simulation data.

### Exploratory exposure-response evaluation for bleeding endpoint

To explore the relationship between dabigatran PD parameters and clinically-related bleeding events in patients with AF, we aimed to identify the best biomarker to predict bleeding events. In the exposure-response analysis, we used subject-specific predictions of PD marker steady-state peaks and troughs obtained from empirical Bayes predictions. We assessed the bleeding endpoint as a binary outcome (yes/no). Besides, we generated a PK/PD box diagram stratified by bleeding events. When we observed exposure-response trends, we performed further analyses using a linear logistic regression model to obtain the significance of exposure after incorporating baseline covariates as potential predictors of event probability.

### Model-based simulation

We evaluated the effects of dose and individual covariates on PD parameters using the final population PK/PD model. We repeated each simulation 1,000 times. We evaluated the simulated PD parameters by calculating steady-state concentrations, peaks, troughs, and the associated 95% confidence intervals (CI).

## Results

### Participant characteristics

We included a total of 118 healthy volunteers and 167 patients with NVAF in our model development process. Table 1 shows the baseline characteristics of all the participants. The healthy subjects all received a single dose of 150 mg dabigatran etexilate, either under fasting (*n* = 59) or fed (*n* = 59) conditions. The total number of PK, APTT, PT, and anti-FIIa observations in the healthy group were 1,849, 472, 468, and 447, respectively. Patients with NVAF generally received dabigatran etexilate 110 mg, twice daily under fasting (*n* = 143) or fed (*n* = 24) conditions. The total number of APTT, PT, and anti-FIIa observations in patients were 329, 330, and 331, respectively. Most indexes were significantly different between healthy subjects and patients. Additionally, patients had a much lower CrCL than healthy volunteers (64.48 vs. 120.18 mL/min, *p* < 0.001).

**Table 1:** Baseline characteristics of subjects included in the model.

**Variables**	**Healthy volunteers**	**Patients**
*n*	118	167
Total number of PK observations	1,849	–
Total number of APTT observations	472	329
Total number of PT observations	468	330
Total number of Anti-IIa observations	447	331
Dose, mg (*n*) and regimen	150 mg (*n* = 118), single dosing	110 mg(*n* = 167), twice dosing
Fed or fasted, *n* (%)	
Fed	59 (50.00%)	143 (85.63%)
Fasted	59 (50.00%)	24 (14.37%)
Male or Female, *n* (%)	
Male	85 (72.03%)	104 (62.28%)
Female	33 (27.97%)	63 (37.72%)
Age, years	22 (18–41)	73 (39–91)
Body height, cm	165.5 (146–180.5)	168 (150–186.5)
Body weight, kg	60.3 (50.3–73.8)	70 (44–115)
Body mass index, kg/m^2^	22.1 (19.1–25.9)	24.7 (16.7–46.6)
Creatinine, umol/L	68 (38–97)	81.55 (37–159)
ALT, U/L	16 (4–49)	19 (5–130.3)
AST, U/L	20 (9–39)	20 (8.4–228)
ALP, U/L	63 (25–131)	66.25 (24–235)
HGB, g/L	151 (118–175)	135 (43.5–182)
TG, mmol/L	0.79 (0.36–2.19)	1.13 (0.37–3.48)
TCHO, mmol/L	4.26 (0.3–6.17)	3.59 (1.74–8.05)
LDL, mmol/L	2.46 (1.17–4.39)	2.08 (0.5–6.43)
HDL, mmol/L	1.42 (1.02–2.5)	1.04 (0.42–2.43)
CRCL, ml/min	120.18 (86.96–171.02)	64.48 (23.00–179.55)

Annotation: *n*, number of subjects/patients; PK, pharmacokinetics; APTT, activated partial thromboplastin time; PT, prothrombin time; ALT, alanine transaminase; AST, aspartate transaminase; ALP, alkaline phosphatase; HGB, hemoglobin; TG, triglyceride; TCHO, total cholesterol; LDL-C, low-density lipoprotein; HDL-C, high-density lipoprotein; CrCL, creatinine clearance. Data are presented as median (range) unless stated otherwise.

### PopPK and PK/PD modeling analysis of healthy subjects

#### PopPK model

In line with a previous dabigatran etexilate concentration modeling study, we found that a two-compartment linear model with first-order absorption best describes the PK of dabigatran etexilate (Supplementary Figure 1) (14, 15, 24).

Due to the lack of estimation data, we fixed the relative bioavailability at 1. Besides, based on the literature, we fixed the lag time at 0.634 h. We estimated the absorption coefficient to 0.261 L/h and the Cl/F to 166 L/h. Finally, we estimated the central ventricular apparent distribution volume and peripheral ventricular apparent distribution volume to 235 and 345 L, respectively. The IIV of Cl/F decreased from 18.7 to 3.7% after we included the covariates of HDL-C and age. The additive residual variability of the model was 221 ng/ml. Besides, the shrinkage rate η was reasonable (< 50%). Table 2 shows the final overall estimates of the PK parameters, Supplementary Table 3 displays the population pharmacokinetic modeling process, Supplementary Figure 2 displays the GoF diagram with overall prediction and individual prediction of PK parameters in healthy subjects, and Supplementary Figure 3 presents the pcVPC of the PopPK model in healthy subjects. Overall, the PK model predictions are consistent with the observed dabigatran etexilate data.

**Table 2:** Final population estimates for parameters of healthy volunteers and patients in the integrated model.

**Parameter**	**Healthy volunteers model**		**Integrated model**	
	**Estimates (%RSE)**	**IIV (%RSE)**	**Estimates (%RSE)**	**IIV (%RSE)**
PopPK				
CL/F (L/hr)	166 (3.7%)	0.142 (15.4%)	166, FIX	–
Q/F (L/hr)	35.5, FIX	–	35.5, FIX	–
V2/F (L)	235 (11.4%)	1.14 (17.3%)	235, FIX	1.14, FIX
V3/F (L)	345, FIX	–	345, FIX	–
Ka (1/hr)	0.261 (3.3%)	0, FIX	0.261, FIX	0, FIX
ALAG1 (hr)	0.634, FIX	0, FIX	0.634, FIX	0, FIX
F1 (hr)	1, FIX	0, FIX	1, FIX	0, FIX
Impact of HDL on CL/F	0.694 (33.0%)	–	0.694, FIX	–
Impact of AGE on CL/F	0.506 (37.4%)	–	0.506, FIX	–
Additive residual error ( ng/ml)	221 (11.4%)	–	221, FIX	
APTT				
E0 (s)	30.4 (1.3%)	0.0144 (22.5%)	32 (1.7%)	0.0258 (17%)
K1	0.0911 (7.4%)	–	0.0643 (17%)	–
Impact of AGE on E0	–	–	0.0381 (27%)	–
Impact of CRCL on E0	–	–	0.252 (12.2%)	–
Additive residual error	30.1 (9.7%)	–	0.0258 (17%)	–
PT				
E1 (s)	11.7 (0.5%)	–	11.8 (0.6%)	–
K2	0.00836 (9.2%)	0.0215 (34.7%)	0.006 (16.2%)	1.25 (20.4%)
Impact of CRCL on K2	–	–	2.02 (8.8%)	–
Additive residual error	0.592 (7.8%)	–	0.89 (9.9%)	–
Anti-IIa				
E2	14.9 (10.7%)	–	20.5 (9.3%)	–
K3	0.628 (8.5%)	0.257 (24.9%)	0.562 (11.7%)	1.1 (17%)
Impact of CRCL on K3	–	–	1.35 (13.8%)	
Additive residual error	643 (11.5%)	–	1,190 (10.3%)	

#### PK/PD modeling

##### APTT model

A linear direct effect model with additive error best describes the relationship between APTT and dabigatran plasma concentration. Table 2 sums up the PD modeling results of healthy volunteers. In this study, the baseline value of APTT (E0) is 30.4 s, and the slope of the correlation between APTT and dabigatran etexilate concentration (K) is 0.0911 s.mL.ug^−1^. The IIV was low, the baseline IIV was 1.44%, and the slope IIV was fixed at 0. In the analysis, we found no covariates significantly impacting E0. The residual (additive) variability remaining in the model is relatively low at 30.1 s.

##### PT model

A linear direct effect model with additive error best describes the relationship between PT and dabigatran plasma concentration. Table 2 displays the PD modeling results of healthy volunteers. In this study, the baseline value of PT (E0) was 11.7 s, and the correlation slope between PT and dabigatran etexilate concentration (K) was 0.00836 s.mL.ug−1. The IIV was low, the baseline IIV was fixed at 0, and the slope IIV was 2.15%. In the analysis, we found no covariates significantly impacting K. The residual (additive) variability remaining in the model is relatively low at 0.592 s.

##### Anti-FIIa model

A linear direct effect model with additive error best describes the relationship between anti-FIIa and dabigatran plasma concentration. Table 2 exhibits the PD modeling results of healthy volunteers. The baseline value of anti-FIIa (E0) was 14.9 ng/ml, and the correlation slope between anti-FIIa and dabigatran etexilate concentration (K) was 0.628 ng^−1^.ml.ug^−1^. IIV was moderate, the baseline IIV was fixed at 0, and the slope IIV was 25.7%. In the analysis, we found no covariates significantly impacting K. The residual (additive) variability remaining in the model was relatively low at 643.

### Integrated PK/PD model

Since PK data of patients were scarce (23 cases), we used their PK data for pcVPC external verification of the PK model of healthy subjects. As Supplementary Figure 3 shows, the PK model of healthy subjects is also applicable to patients.

In the modeling step, we fixed the PK a priori parameters of healthy subjects and patients (such as absorption coefficient, relative bioavailability, lag time, Cl, VC, Q, and VP) because the patient data were not included in the establishment process of the comprehensive model. In the comprehensive model, the covariates CrCL and age of the fixed parameter CL/F in the PK model remain unchanged, and the additive residual variability of the model remains unchanged, recalculate the residual variation in the PD model to explain the variability between different populations.

**Figure 1:**
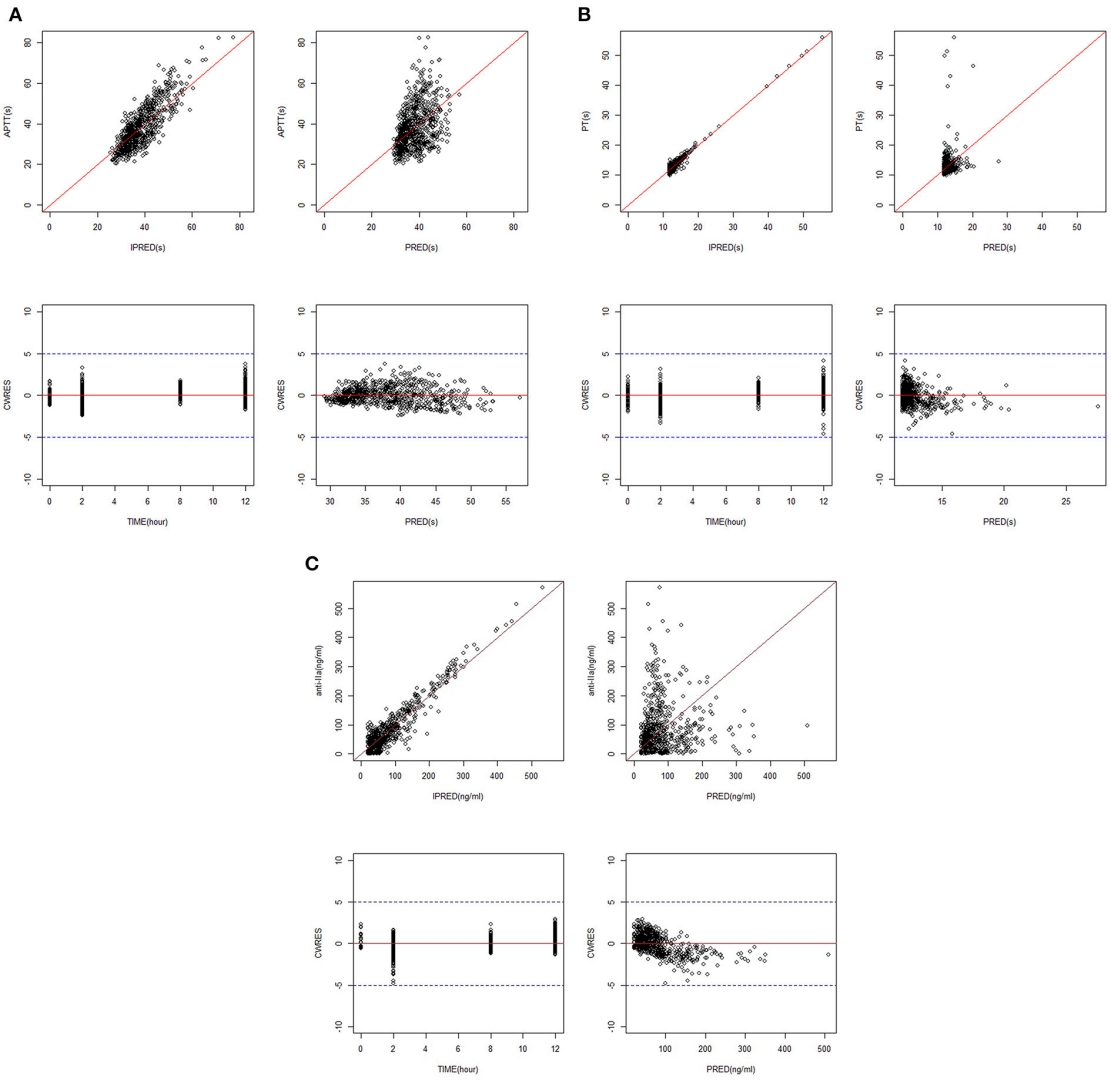
GoF plots for the integrated PK/PD model. **(A)** APTT. **(B)** PT. **(C)** Anti-FIIa. Annotation: The standard goodness-of-fit plots for APTT, PT, and anti-FIIa in the integrated PK/PD model.

**Figure 2:**
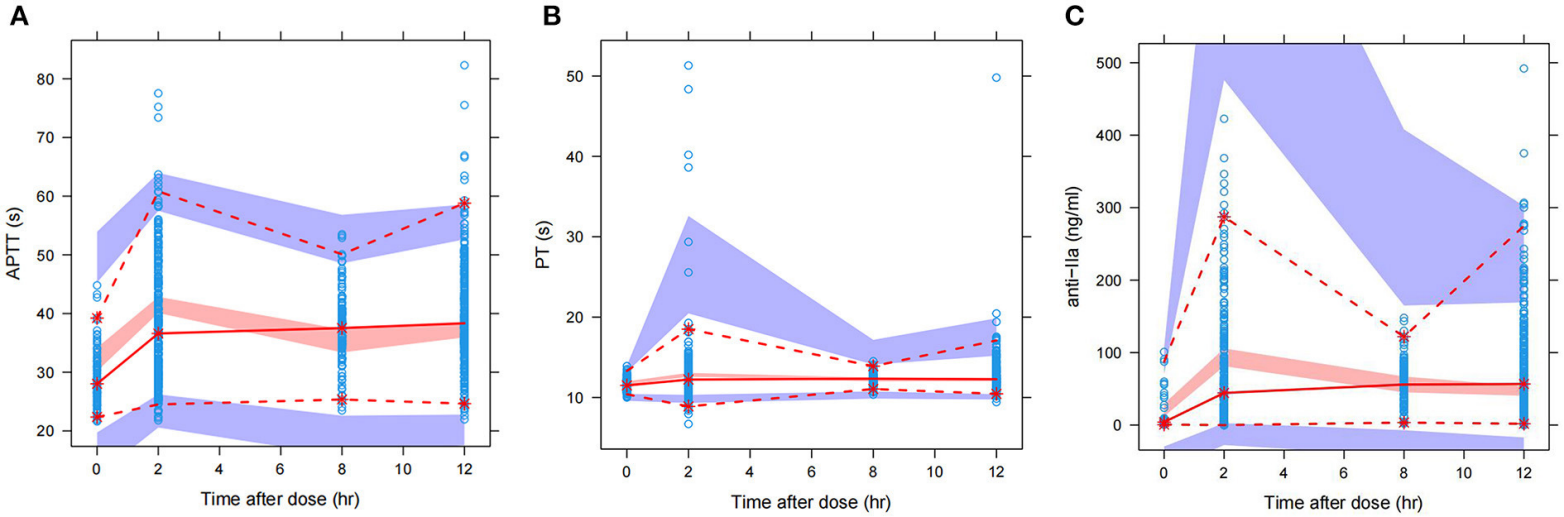
pcVPC of the integrated PK/PD model. **(A)** APTT. **(B)** PT. **(C)** Anti-FIIa. Annotation: The prediction-corrected visual prediction test (pcVPC) for APTT, PT, and anti-FIIa in the integrated PK/PD model.

Figures 1A–C shows the GoF diagram of the overall prediction and individual prediction of the integrated APTT, PT, and anti-FIIa model. The population prediction, individual prediction, and dependent variable diagram of each model are evenly distributed around the 1:1 line. Conditional weighted residuals are randomly distributed around zero in population prediction value and time. Figure 2 shows the pcVPC verification results of the integrated PD model. The observed values of each model are randomly distributed in the calculated 95% prediction interval, indicating a good overall consistency between the final model and the observed data.

### Exploratory exposure-response analysis

**Figure 3:**
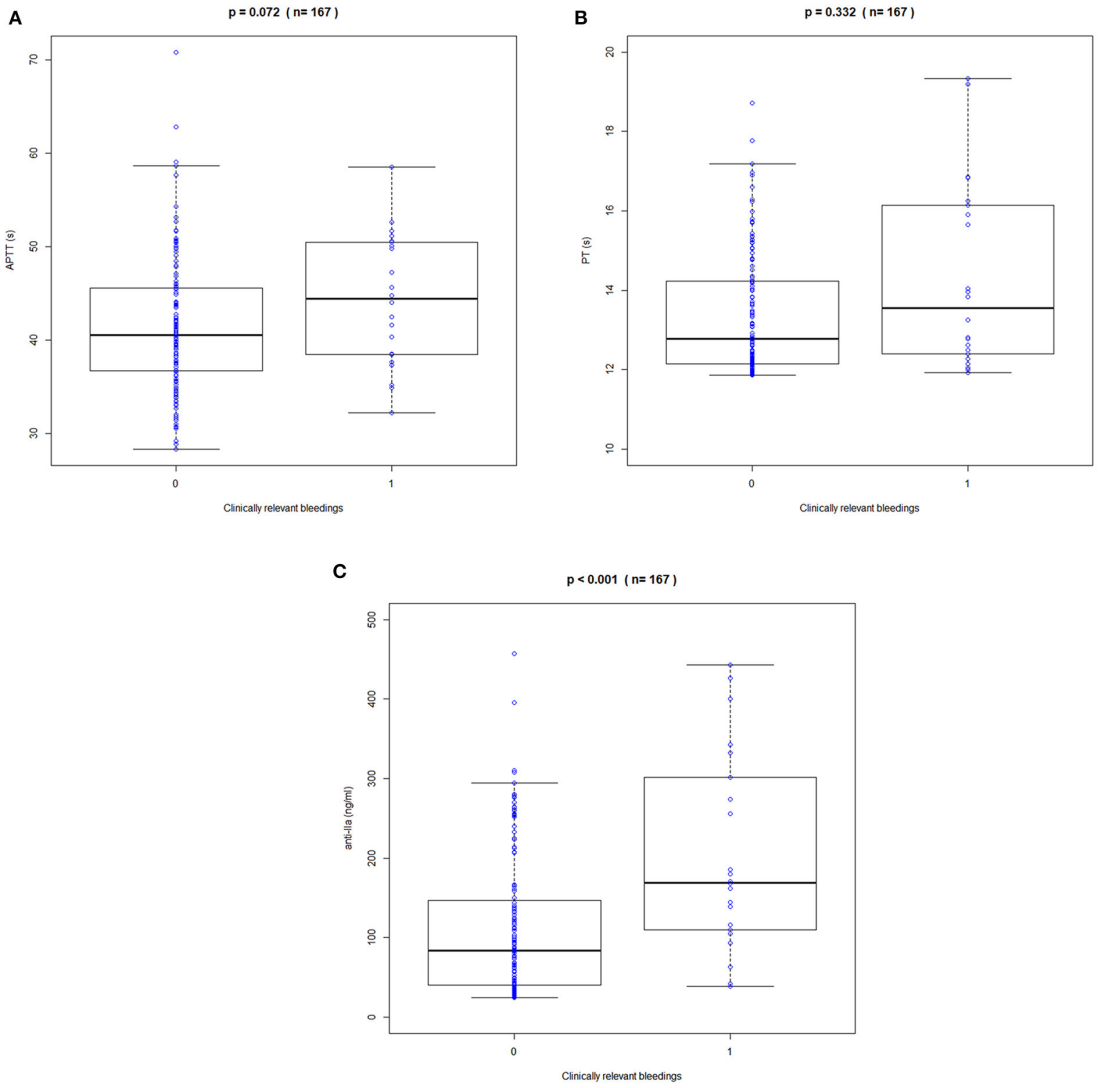
Correlation between peak APPT, PT, and anti-FIIa values and bleeding events. **(A)** APTT. **(B)** PT. **(C)** Anti-FIIa. Annotation: The blue circle represents the standard dose. Boxes indicate the 25th−75th percentiles, whiskers represent 1.5 times the interquartile range, and black horizontal lines represent the median. The blue circles are individual predicted values for the standard dose or overdose.

**Figure 4:**
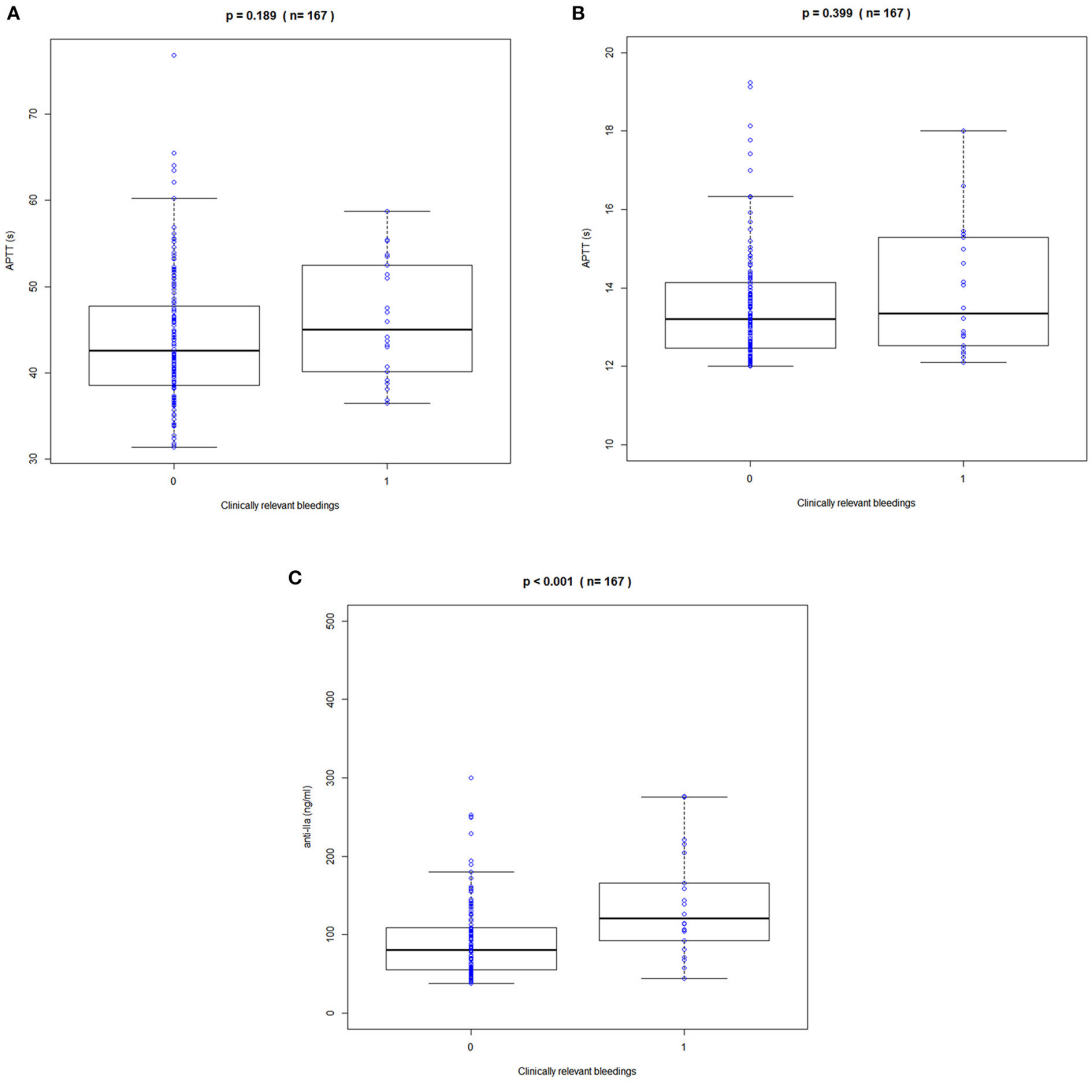
Correlation between trough APPT, PT, and anti-FIIa values and bleeding events. **(A)** APTT. **(B)** PT. **(C)** Anti-FIIa. Annotation: The blue circle represents the standard dose. Boxes indicate the 25^th^-75^th^ percentiles, whiskers represent 1.5 times the interquartile range, and black horizontal lines represent the median. The blue circles are individual predicted values for the standard dose or overdose.

Clinical bleeding events occurred in 22 of 167 patients (13.17%). Patients with bleeding were followed up for 2 years, with a median of 6 months (range 1–24 months). Figures 3, 4 shows the ANOVA box plots of APTT, PT, and anti-FIIa in bleeding and non-bleeding patients. The bleeding group had higher peak values of APTT (51.42 s), PT (14.55 s), and anti-FIIa (227.96 ng/ml) and trough values of APTT (40.44 s), PT (14.81 s), and anti-FIIa factor activity (103.83 ng/ml) than the non-bleeding group. In addition, we calculated the linear logistic regression values of PD parameters and bleeding events and plotted the correlation scatter diagram (Supplementary Figure 4). Finally, cox analysis has been conducted on the correlation between PD indicators (APPT, PT, ANTI) and bleeding risk, but the results have no significant impact (*P* = 0.943, 0.665, 0.966 > 0.5). The variance and regression analyses revealed no clear relationship between clinical endpoints and PD parameters except anti-FIIa.

### Exposure simulation

**Figure 5:**
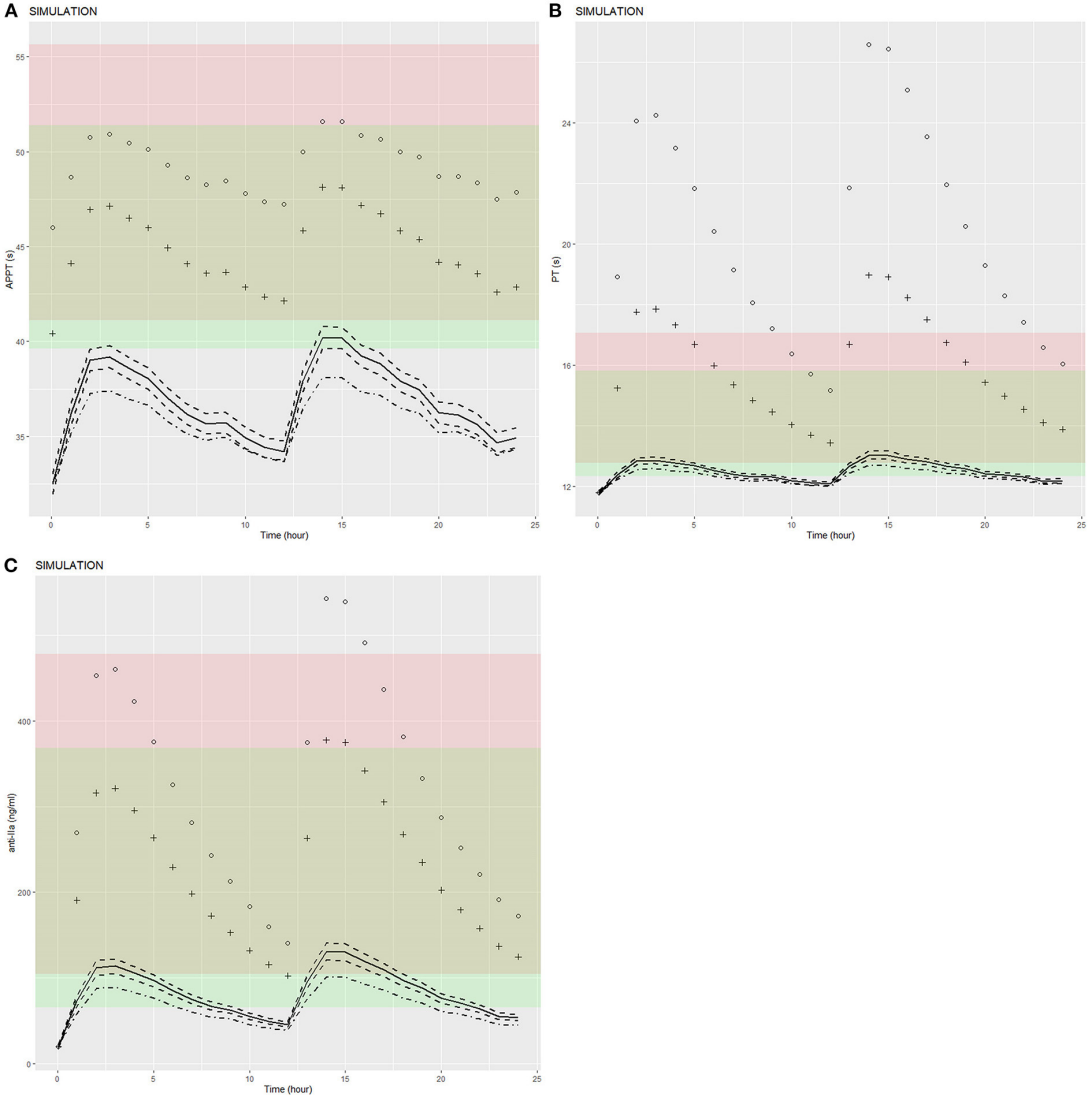
Predicted APTT, PT, and anti-FIIa PD-time profiles. **(A)** APTT. **(B)** PT. **(C)** Anti-FIIa. Annotation: The black dot represents a patient with mean characteristics (dose = 110 mg bid, HDL = 1.4 mmol/L, age = 24 y, CrCL = 30 mL/min). The black cross represents a patient with mean characteristics (dose = 150 mg bid, HDL = 1.4 mmol/L, age = 24 y, CrCL = 50 mL/min). The black solid line represents a patient with mean characteristics (dose = 150 mg bid, HDL = 1.4 mmol/L, age = 24 y, CrCL = 118.76 mL/min) and the black dotted line 1 represents the corresponding 95% confidence interval. The black dotted line 2 represents the 95% confidence interval of a patient with mean characteristics (dose = 110 mg bid, HDL = 1.4 mmol/L, age = 24 y, CrCL = 118.76 mL/min). The red shaded area represents the predicted peak concentration ranges of APTT, PT, and anti-FIIa. The green shaded area represents the predicted trough concentration ranges of APTT, PT, and anti-FIIa. The yellow shaded area represents the predicted overlap of the trough and peak concentration ranges of APTT, PT, and anti-FIIa.

We used the final integrated PK/PD model to predict the PK/PD parameters of each virtual subgroup (defined by HDL-C, age, and CrCl) in the steady state. Figure 5 shows the simulation results of APPT, PT, and anti-FIIa. Using the final PK/PD model, we generated typical patient reference values as the real world. The peak and trough times of PD parameters (APTT, PT, and anti-FIIa) obtained by simulation were 2 h and 0.05 h, respectively. The peak values of APTT, PT, and anti-FIIa after administration of 150 mg of dabigatran etexilate were 40.20 s (95% CI: 39.61–40.78 s), 13.01 s (95% CI: 12.88–13.15s), and 130.58 ng/ml (95% CI: 120.40–140.77 ng/ml), respectively. The trough values of APTT, PT, and anti-IIa were 32.49 s (95% CI: 31.95–33.02 s), 11.79 s (95% CI: 11.73–11.85 s), and 20.13 ng/ml (18.04–22.22 ng/ml), respectively. Unlike APTT, PT and anti-IIa had values within the expected range caused by the standard dose (25). Unlike typical patients, subjects with a CrCl of 30 mL/min had peak values of APTT, PT, and anti-IIa of 51.58 s, 26.59 s, and 543.18 ng/ml, respectively, and trough values of APTT, PT, and anti-IIa factor activities of 45.98 s, 11.79 s, and 20.13 ng/ml, respectively. The recommended dabigatran etexilate dose was 110 mg. Subjects with a CrCl of 50 mL/min had peak values of APTT, PT, and anti-IIa of 48.13 s, 18.97 s, and 377.68 ng/ml, respectively, and trough values of APTT, PT, and anti-IIa of 40.42 s, 11.79 s, and 20.13 ng/ml, respectively. The recommended dabigatran etexilate dose was 150 mg. For both doses, the recommended administration frequencies are the same as in clinical practice, namely twice a day. The peak and trough values of APTT, PT, and anti-IIa in patients who received the 150 mg dose and had a CrCl >50 mL/min were lower than those in patients who received the 110 mg dose and had a 30–50 mL/min CrCl, and could be maintained within the expected range of the standard dose (25). These results indicated that parameters other than dosage and CrCl had little effect on dabigatran exposure.

## Discussion

This study thoroughly explored a PK model of a single group of healthy volunteers and the PD model of healthy volunteers and patients. Due to the relative scarcity of PK samples from patients (23 cases), we cannot directly estimate the PK parameters of patients. However, the PD data collected in this study are relatively rich. We used the few PK data of patients to verify that the healthy subjects PK model is also applicable to patients and searched the relevant literature to confirm our results. We assumed that the differences observed in this study mainly come from the progress of PD; thus, the variation between populations can be explained by recalculating the parameters of the PD model. In our study, the average ages of healthy subjects and patients with NVAF were 22 and 73 years, respectively. The median CrCl of patients with NVAF was 40.92% lower than that of healthy subjects, and patients had higher trough values than healthy subjects for APTT (39.3 vs. 34.1 s), PT (13.9 vs. 11.9 s), and anti-FIIa (67.17 vs. 38.7 ng/ml), in line with a comparative study on the PK of rivaroxaban in young and older people (26–28). Therefore, we expect CrCL, the mixed effects of creatinine, weight, age, and gender, calculated using the CG formula (29), to affect the PK of dabigatran.

To enrich and stabilize the structural model without changing it, we pooled data from healthy volunteers and patients. In addition, due to the overall scarcity of PK data of healthy volunteers and patients, we could not accurately estimate the peripheral ventricular clearance rate and peripheral ventricular apparent distribution volume and needed to fix them at the typical values of the population. Meanwhile, due to the lack of data on the absorption stage, we fixed the individual variation of relative bioavailability, absorption rate constant, and lag time at 0, which limits the model's capacity to predict the absorption stage. However, based on another DOAC named rivaroxaban, we expected these parameters to have little effect on the derived exposure (26). Furthermore, according to previous clinical pharmacological studies, meal time and content do not seem to affect dabigatran concentration (14, 15, 24). Thus, we successfully developed a comprehensive model by exploring the relationship between the PK and PD of healthy individuals and the PD data of patients.

Finally, in the comprehensive PK/PD model, Cl/F is 166 mL/min, similar to previously reported values (124 and 107 mL/min) (14, 15). Covariate analysis showed that age and HDL-C were the most significant covariates affecting PK parameters, and age and CrCL were the most significant covariates affecting PD parameters. This is mostly in line with expectations (14, 15, 24). Many PK studies identified age as a covariate significantly impacting the PK parameters of dabigatran etexilate (14, 24). Dabigatran etexilate is a precursor drug, which is transformed into the active product dabigatran through the catalysis of carboxylesterase to produce pharmacological effects. At the same time, carboxylesterase has also been proved to affect lipoprotein. Joseph (25, 26) found that dabigatran ester can reduce serum apolipoprotein B, the main component of low density lipoprotein (LDL). Kose (29) found that HDL has a significant impact on patients with prolonged or normal APTT of dabigatran axetil. It can be further assumed that HDL is a potential influencing factor of APTT of dabigatran axetil. The PK/PD model of dabigatran ester APTT established in this study reveals the correlation between concentration and APTT, so the influence of HDL on concentration can be explained theoretically. Finally, although there are few studies on the relationship between dabigatran ester and lipoprotein (25, 26, 29), it is still confirmed that dabigatran ester has an indirect impact on lipoprotein metabolism. Pharmacological studies abroad have mainly revealed its impact on low-density lipoprotein (25, 26), but considering the differences in race and pharmaceutical preparation process, it is still possible that it may have an impact on high-density lipoprotein. Meanwhile, this study is the first to point out the significant influence of covariates such as age and CrCl on the PD parameters of dabigatran etexilate (15).

Because our study has a large PK dataset from healthy volunteers, the PK model of healthy volunteers is relatively robust. Age and HDL-C reduce the IIV of dabigatran etexilate absorption, thus improving the predictability of PK; they were thus retained in the final PK model.

The final PD model is a general linear model (with a baseline value, E0, and a slope, K) using a direct effect model, similar to a previous model (14, 15). It is worth noting that our model also shows that CrCL negatively affects the slope of APTT, PT, and anti-FIIa, indicating that the increase rate of the PD index is less than that of plasma concentration, although the reason for this has not been determined. The estimated mean slope (APTT = 0.0643 s.mL/μg, PT = 0.006 s.mL/μg, anti-FIIa = 0.562 ng/μg) showed that anti-FIIa was more sensitive to the increased exposure to dabigatran than APTT and PT were, which was consistent with the GoF diagram. This observation suggests that anti-FIIa activity can more sensitively reflect dabigatran etexilate exposure in clinical decision-making, for example in overdose and emergency surgery. This finding is consistent with the evidence-based guidelines of the International Committee for Hematological Standardization (ICSH), which recommends the anti-FIIa test for quantitative oral direct FIIa inhibitors (28).

A small number of bleeding events occurred in our study (22/167 cases, 13.17%), variance analysis, logistic regression and COX regression were used to evaluate the correlation between PD indicators and bleeding risk, but the exposure-response model of bleeding could not be accurately described. However, the bleeding tendency is increased, and the peak values of APTT, PT, and anti-FIIa are increased. In addition, the peak value of anti-FIIa was significantly correlated with bleeding events, indicating that anti-FIIa activity is more worthy of clinical attention than the other PD indicators. Relevant studies also showed that patients with high peak values of PD indicators such as APTT experienced significantly more bleeding events (30). Nevertheless, the trough value is more related to the bleeding risk than the peak value (31). Therefore, identifying the best bleeding risk predictor between the trough or peak value of PD parameters requires a study with a larger sample size.

In order to evaluate the PK model, The drug concentration of dabigatran under the conditions of CRCL of 30 ml/min, dosage of 150 and average age of 75 years was simulated, and the results were similar to those in literature (32) without significant difference. When the simulated administration scheme was 150 mg bid, the median trough and peak values of simulated concentration were 48.68 ng/ml (12 h) and 168.22 ng/ml (14 h) respectively, and the trough and peak concentrations in the literature (32) were 93 ng/ml (10–90% PI: 39.8–215 ng/ml) and 184 ng/ml (10–90% PI: 74.3–383 ng/ml) respectively. The median value of the simulated concentration is within the range of 90% PI of the literature concentration, which is basically consistent with the literature research data.

In this study, we used the advantages of pharmacological analysis to simulate the distribution of PD indicators in typical Chinese patients by using the above data and some supplementary data to identify potential influencing factors. For typical patients, Typical patients who had received 150 mg of dabigatran had PT and anti-FIIa peak and trough values within the expected range after a standard dose (33), excepted APTT values. APTT values deviate from the expected range because subjects and patients have different peak and trough values for the relevant indicators; thus, the distribution similarity of APTT indicators in the two populations is low, resulting in poorer simulation accuracy than for PT and anti-FIIa activity. In addition, the median of the above PD indicators is within the expected range caused by the standard dose. By identifying the covariates that affect PK and PD, several cases with different extreme covariates with correlation can be simulated. Assigning extreme values to parameters yielded little difference in PD indicators, which is consistent with previous studies suggesting that dose adjustment should not be performed except for CrCl differences (16).

Worried about bleeding, doctors in Asia tend to prescribe low-dose new oral anticoagulants (10). In our study, some patients were also asked to reduce the dose. Our simulation results show that, except for APTT, the 150 and 110 mg doses yield similar PD parameters value distributions and peak and trough values within the expected range (33). We recommend that Chinese patients who had a CrCL 50 mL/min use a dose of 150 mg, based on model simulated PD distribution. The 110 mg dose group had significantly lower PD parameters values than the 150 mg dose group. The 150 mg dose yields PD parameters values within the expected range, except for APTT. Dabigatran etexilate reduces the incidence of stroke/systemic embolism more strongly than warfarin and shows good safety; therefore, it may benefit patients more.

We acknowledge that our study has some limitations. First, the pcVPC of the integrated model shows that the overall prediction performance of the final model is acceptable, but the prediction of low anti-FIIa concentrations is insufficient. Given the inherent loss of precision and accuracy of laboratory analyses at low concentrations, these may not be of clinical significance. Our study is also limited by the insufficient collection of blood samples from patients for dabigatran concentration determination. Although we used the sparse data from patients (23 cases) to verify that the PK model developed from healthy subjects' data could predict PK parameters of patients, this process lacks accuracy. In future research, more samples of patient PK should be collected and added to the modeling process to increase the efficiency of patient PK parameters prediction. It is reported in the literature (34) that P-glycoprotein plays a role in the transport of thyroid hormone. Thyroid function may reflect the activity of P-glycoprotein. The exclusion criteria of this study population did not determine the exclusion of patients with abnormal thyroid function, which is one of the limitations and should be improved in further research. The small number of bleeding events (22 cases) seemed to indicate that APTT, PT, and anti-FIIa increased, but this result should be interpreted cautiously. Of course, larger studies should confirm the clinical significance of APTT, PT, and anti-FIIa. Based on the PK and PD data of healthy Chinese subjects and real patients with NVAF, we established a comprehensive PK/PD model of dabigatran in the two populations. We used the final comprehensive PK/PD model to simulate the distribution of the reference biomarkers APTT, PT, and anti-FIIa in Chinese patients with NVAF taking dabigatran etexilate. We demonstrated that the individual predicted values for typical patients taking a 150 mg dose were within the expected range recommended by the guidelines. Covariates other than age, CrCL, and HDL-C have little impact on the comprehensive model. Patients taking a dose of 110 and having a CrCL of 30 mL/min should pay attention to the risk of bleeding.

## Data availability statement

The original data presented in the study are included in the article/Supplementary material, further inquiries can be directed to the corresponding author.

## Ethics statement

The studies involving human participants were reviewed and approved by the Ethics Committee of Peking University First Hospital and all the participating subcentral hospitals. The patients/participants provided their written informed consent to participate in this study.

## Author contributions

QX and Y-mC designed the research. Q-fX, Z-yL, ZW, G-yM, Y-tZ, Z-nZ, D-dY, L-pG, NW, JX, and H-tS performed experiments and collected data. Y-oL, Q-fX, Z-yL, and ZW analyzed the data. Y-oL and Q-fX wrote the manuscript. JJ, QX, and Y-mC participated in the discussion of the results. All authors have read and approved the final manuscript.

## Funding

This study was supported by grants from the National Key R&D Program of China (2016YFC0904900), National Science and Technology Major Projects for Major New Drugs Innovation and Development (2017ZX09101001), National Natural Science Foundation of China (82073935, 81973395, and 81872940), and Beijing Municipal Commission of Science and Technology of China Pharmaceutical Innovation Cultivation and Industry Support Platform Capacity Construction Project (Z191100007619038).

## Conflict of interest

The authors declare that the research was conducted in the absence of any commercial or financial relationships that could be construed as a potential conflict of interest.

## Publisher's note

All claims expressed in this article are solely those of the authors and do not necessarily represent those of their affiliated organizations, or those of the publisher, the editors and the reviewers. Any product that may be evaluated in this article, or claim that may be made by its manufacturer, is not guaranteed or endorsed by the publisher.

## Supplementary material

Supplementary Figure 1 Final structure of the PK/PD model.

Supplementary Figure 2 Goodness-of-fit plot for the PopPK model developed from healthy volunteers data.

Supplementary Figure 3 pcVPC for the PopPK model developed from healthy volunteers and patients data. **(A)** Healthy volunteers, **(B)** Patients.

Supplementary Figure 4-1 Probability plot for clinically relevant bleeding and APTT, PT, and anti-FIIa peak values. **(A)** APTT. **(B)** PT**. (C)** anti-FIIa.

Supplementary Figure 4-2 Probability plot for clinically relevant bleeding and APTT, PT, and anti-FIIa trough values. **(A)** APTT. **(B)** PT**. (C)** anti-FIIa.

Supplementary Table 1 Summary of studies included in the dabigatran PopPK and PK/PD analysis.

Supplementary Table 2 List of CYP3A4 and P-gps potent inducers and inhibitors.

Supplementary Table 3 Population pharmacokinetic modeling process.
