# Supplementary figures and images for: Population pharmacokinetic analysis for dabigatran etexilate in Chinese patients with non-valvular atrial fibrillation
Source: Front Cardiovasc Med. 2022 Oct 28;9:998751. doi: 10.3389/fcvm.2022.998751 (PMC9650305; doi:10.3389/fcvm.2022.998751)

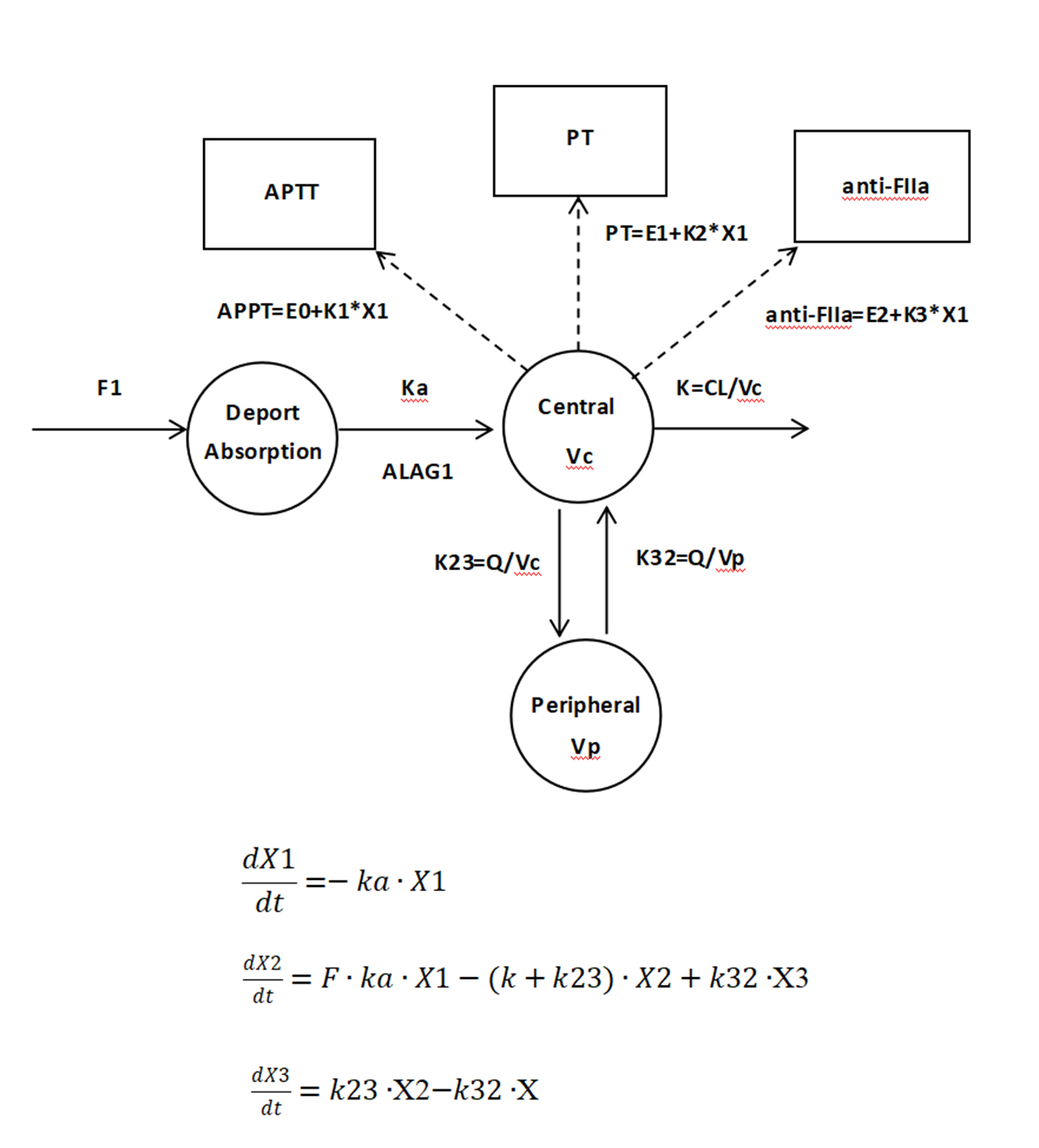

Supplement: Supplementary Figure 1 — Final structure of the PK/PD model. [file Image_1.JPEG]

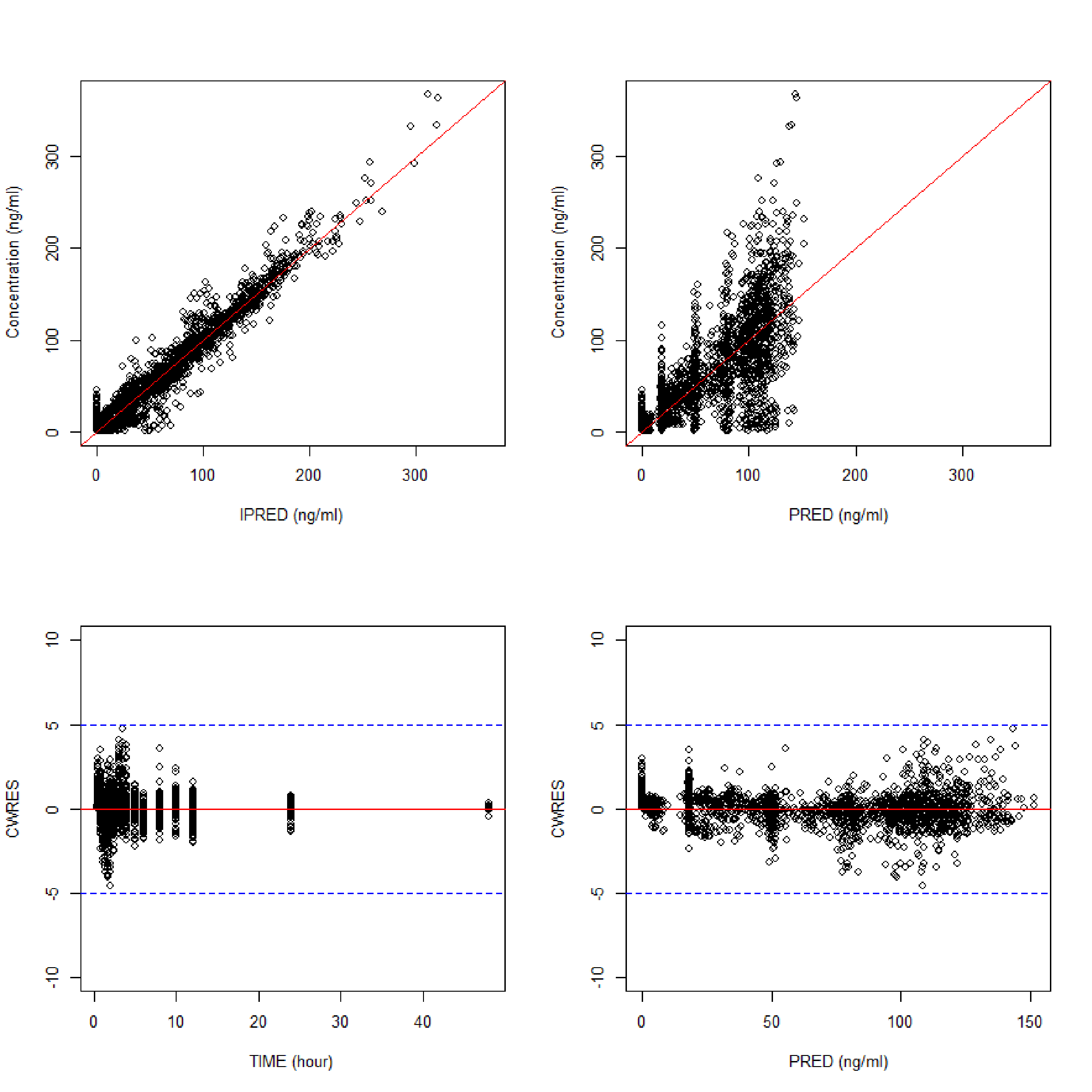

Supplement: Supplementary Figure 2 — Goodness-of-fit plot for the PopPK model developed from healthy volunteers data. [file Image_2.TIF]

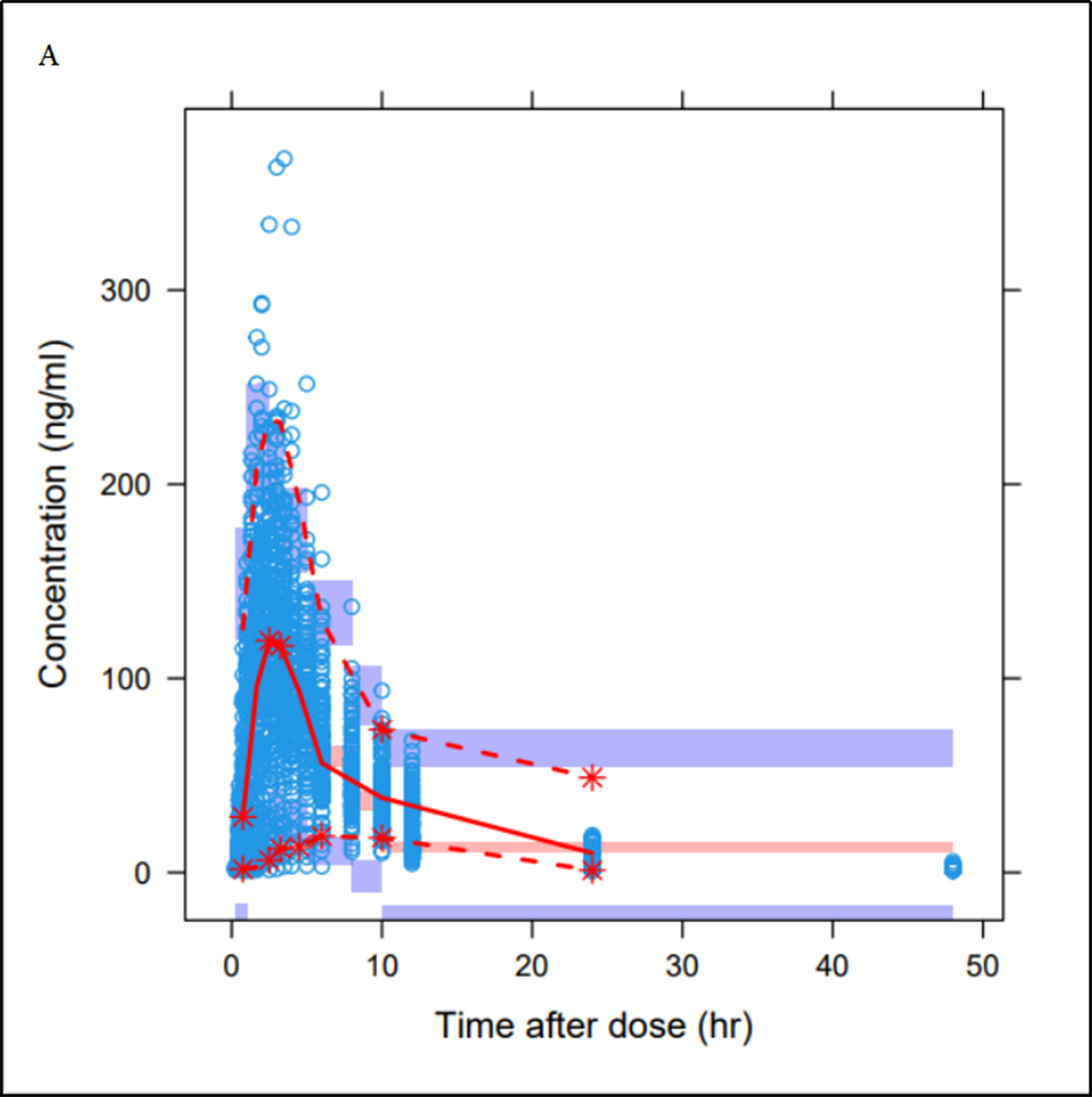

Supplement: Supplementary Figure 3 — pcVPC for the PopPK model developed from healthy volunteers and patients data. (A) Healthy volunteers, (B) Patients. [file Image_3.JPEG]

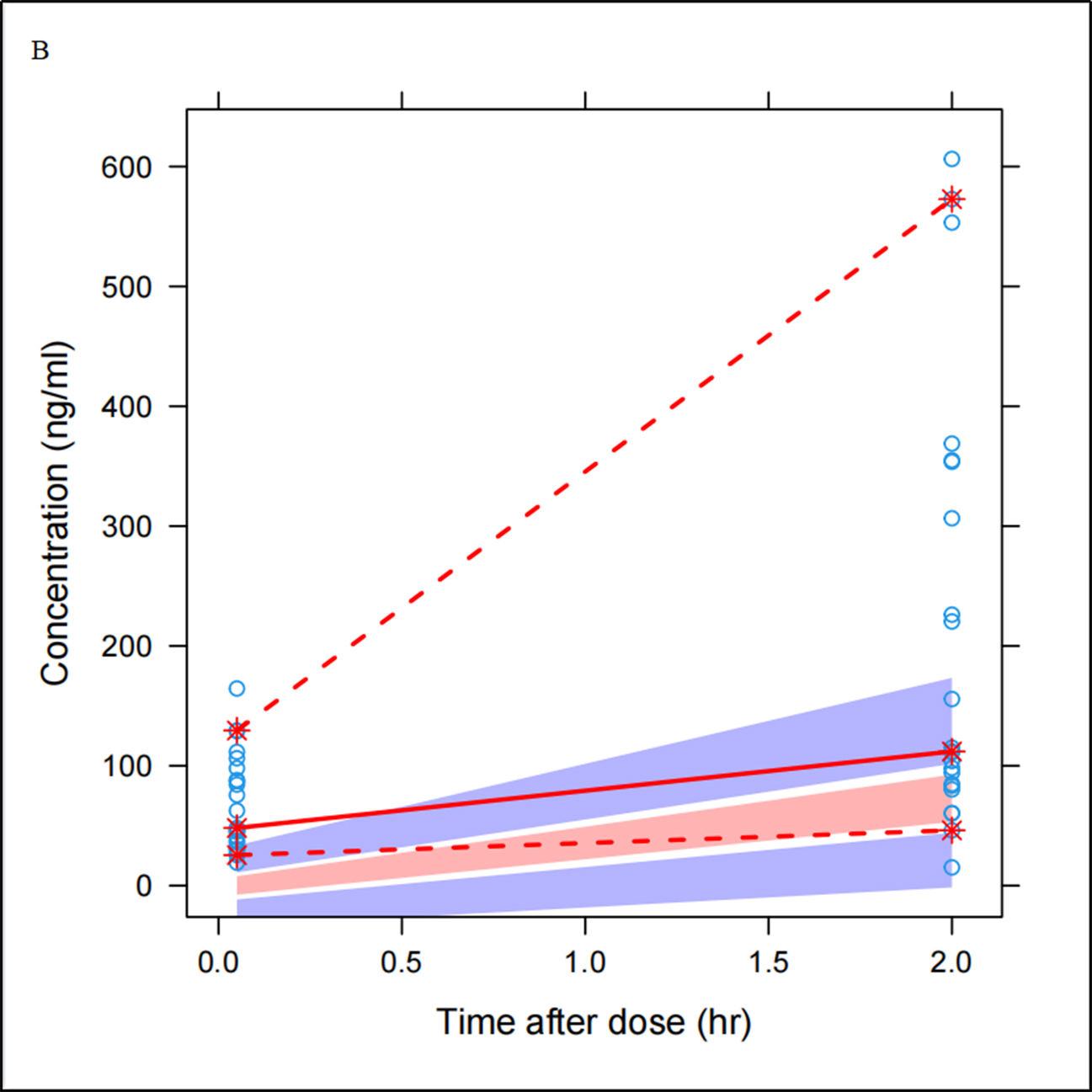

Supplement: Supplementary Figure 4-1 — Probability plot for clinically relevant bleeding and APTT, PT, and anti-FIIa peak values. (A) APTT. (B) PT. (C) anti-FIIa. [file Image_4.JPEG]

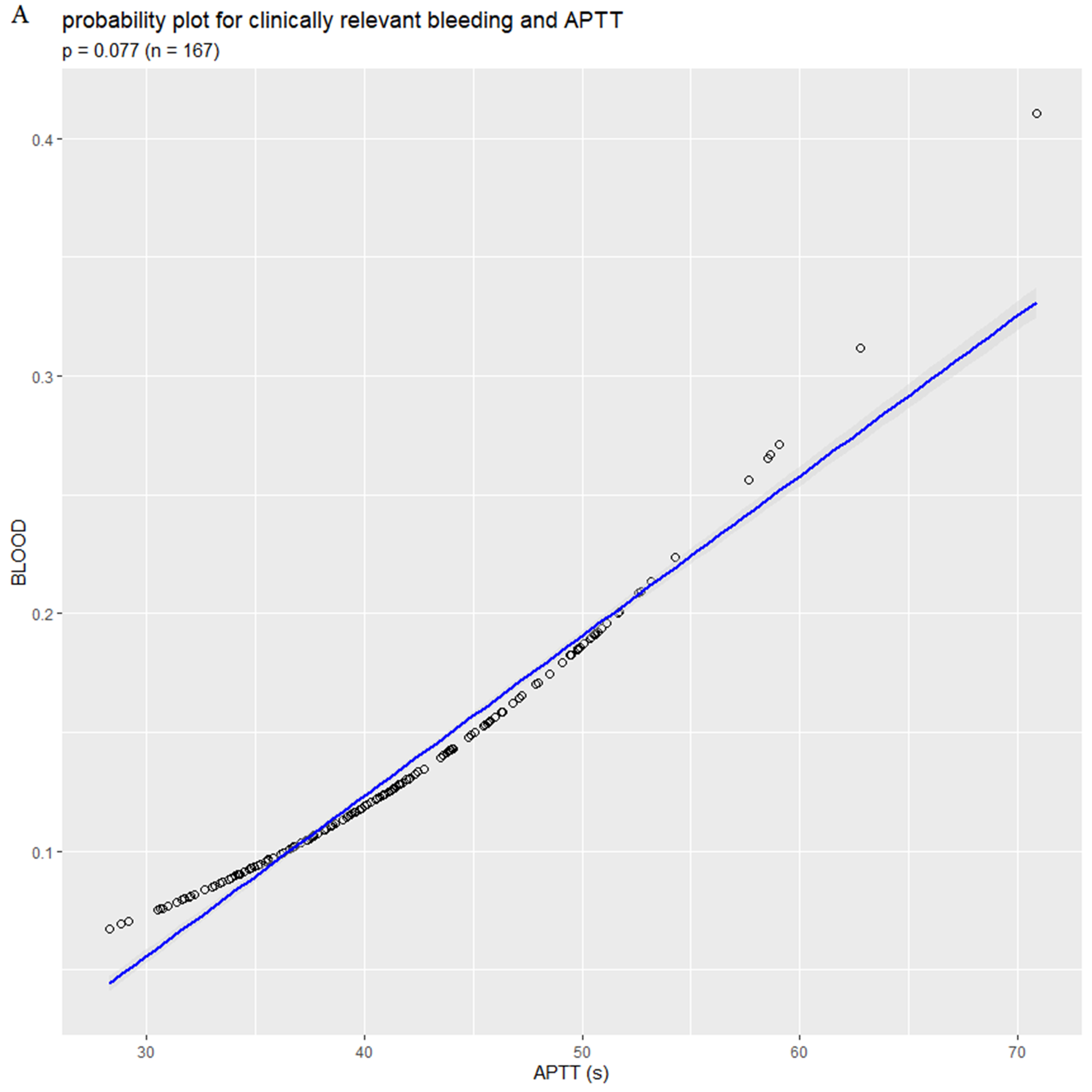

Supplement: Supplementary Figure 4-2 — Probability plot for clinically relevant bleeding and APTT, PT, and anti-FIIa trough values. (A) APTT. (B) PT. (C) anti-FIIa. [file Image_5.JPEG]

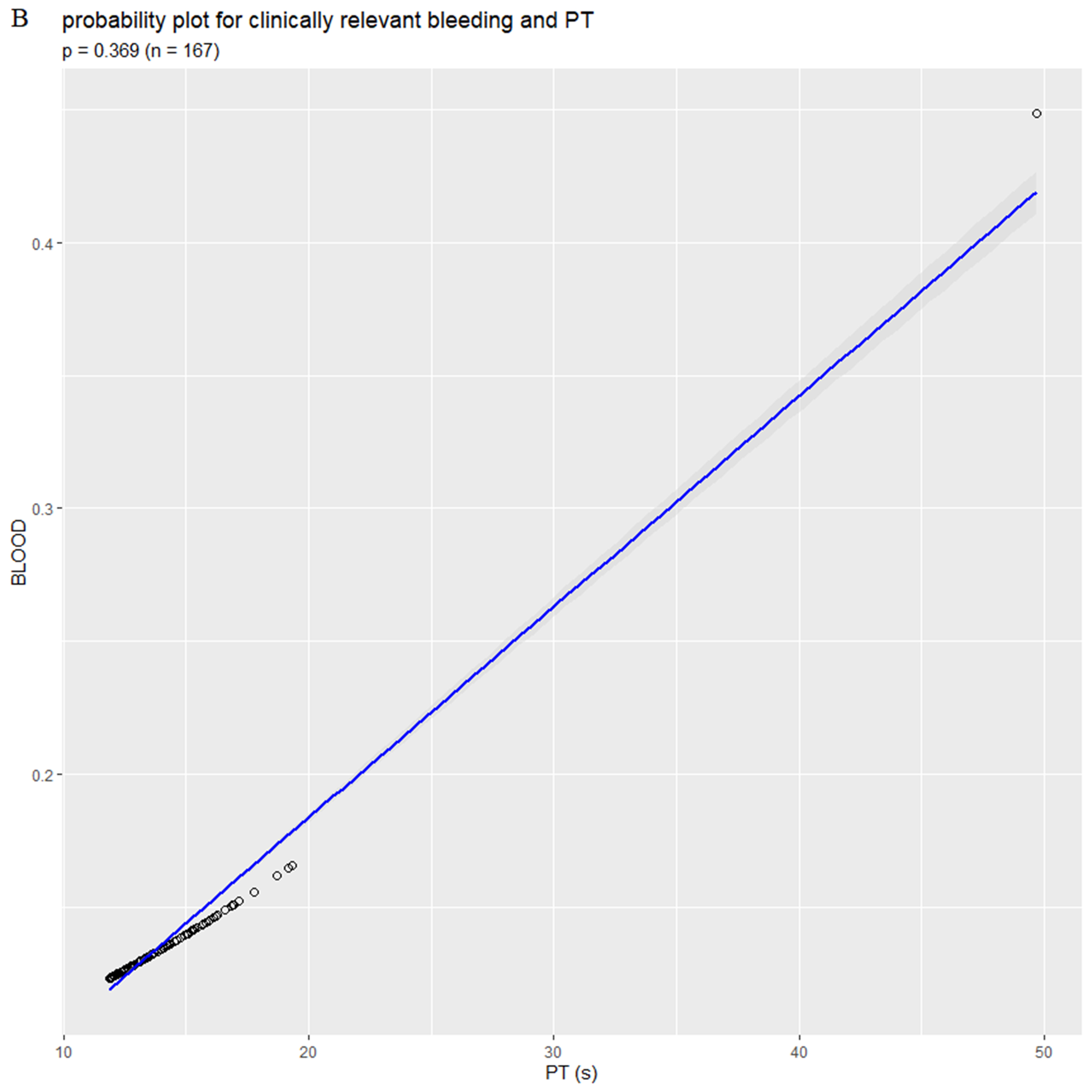

Supplement: Supplementary file 9 [file Image_6.JPEG]

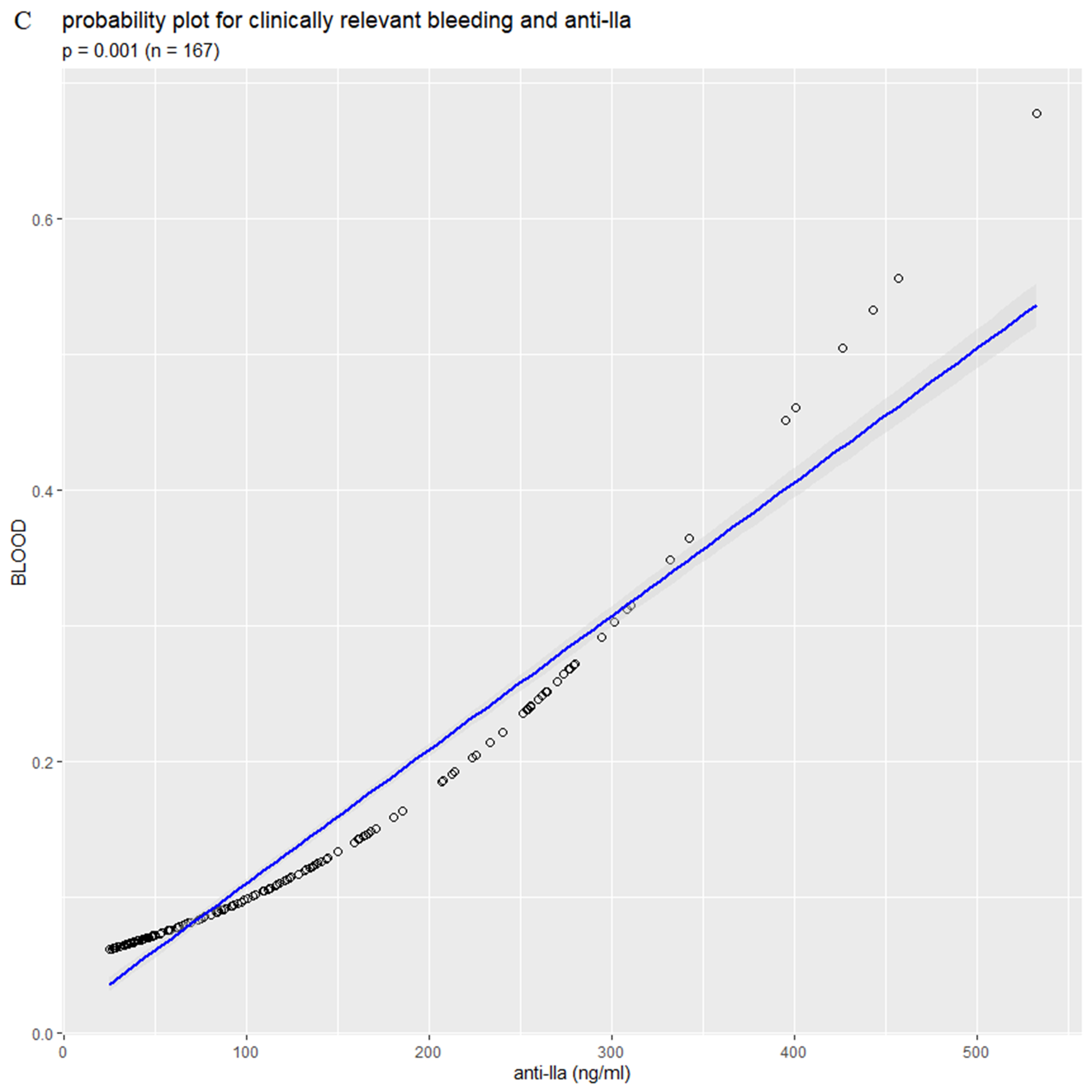

Supplement: Supplementary file 10 [file Image_7.JPEG]

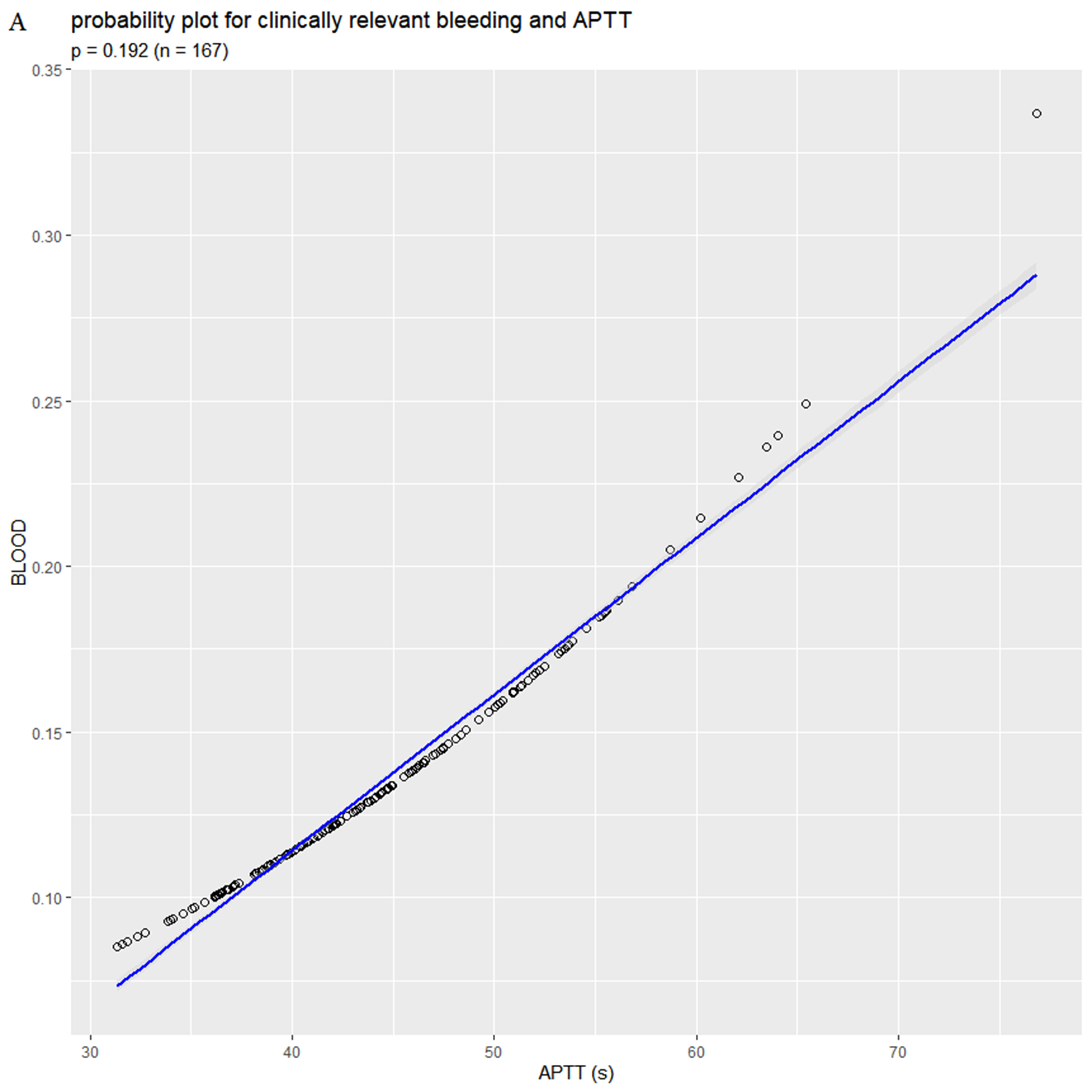

Supplement: Supplementary file 11 [file Image_8.JPEG]

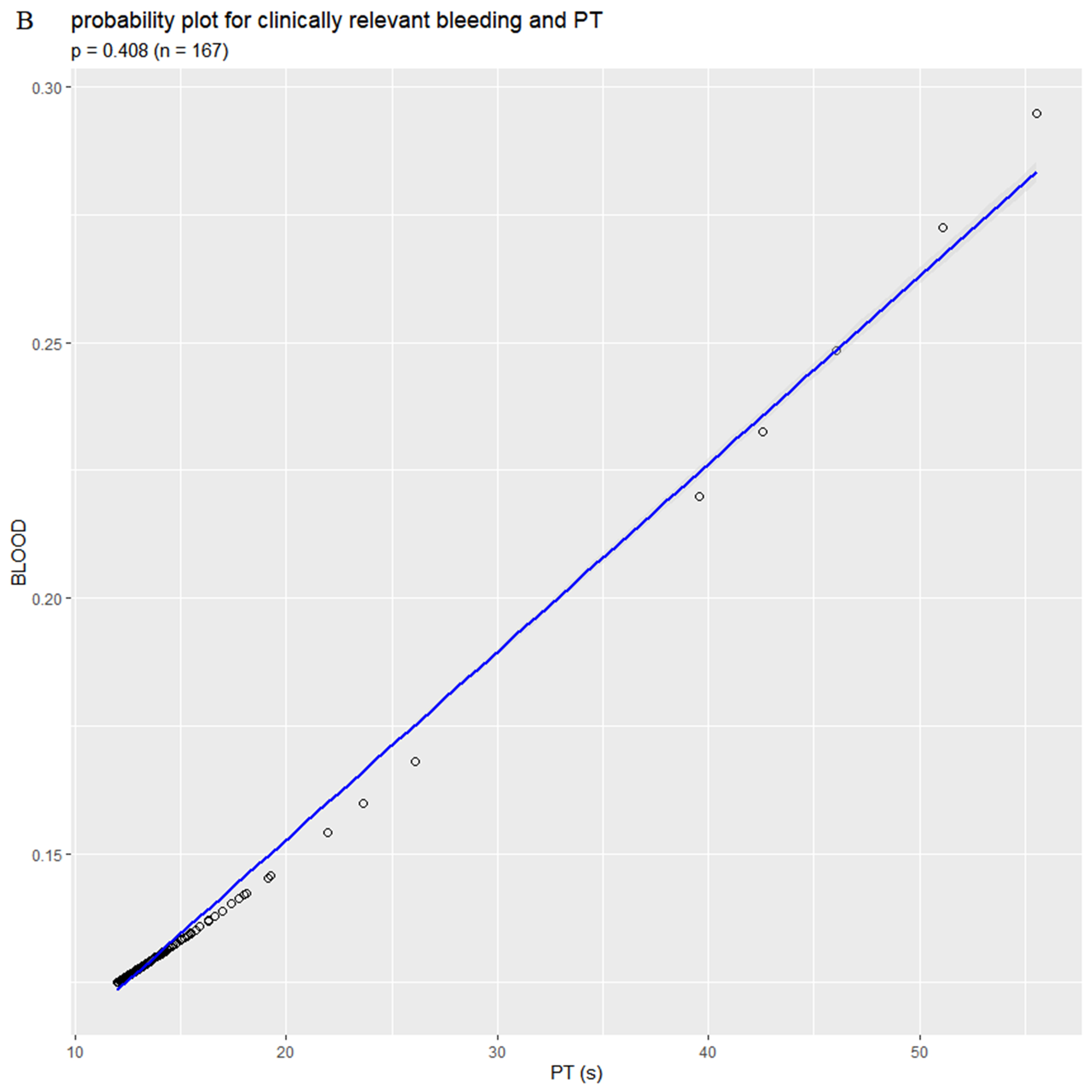

Supplement: Supplementary file 12 [file Image_9.JPEG]

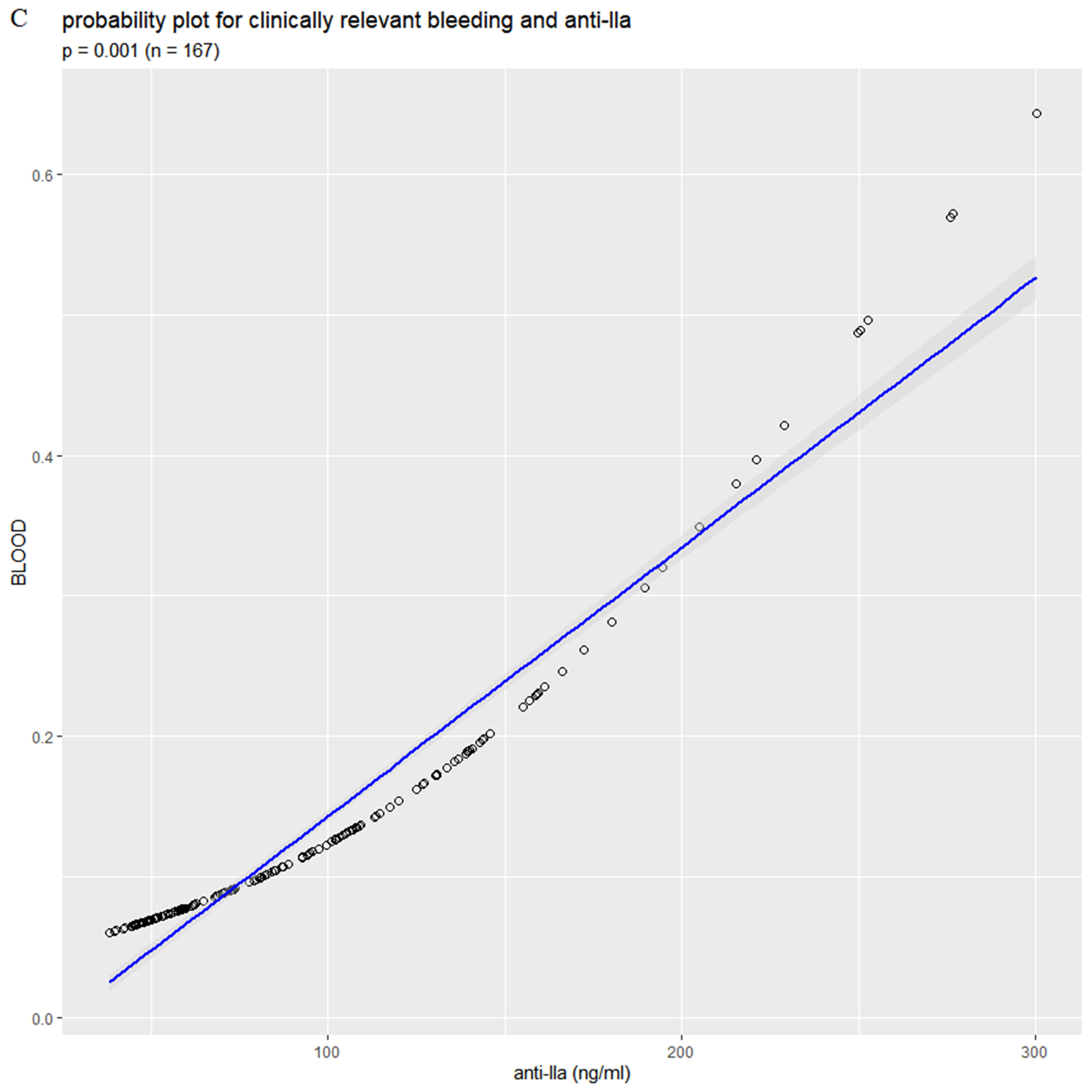

Supplement: Supplementary file 13 [file Image_10.JPEG]
