# Supplementary material for: Population pharmacokinetic analysis for dabigatran etexilate in Chinese patients with non-valvular atrial fibrillation
Source: Front Cardiovasc Med. 2022 Oct 28;9:998751. doi: 10.3389/fcvm.2022.998751 (PMC9650305; doi:10.3389/fcvm.2022.998751)
Supplement: Supplementary Table 1 — Summary of studies included in the dabigatran PopPK and PK/PD analysis. [file Table_1.docx]

Table S1. Summary of studies included in the dabigatran PopPK and PK/PD analysis.

| Study | Objective | Population | N | Ticagrelor  dosing | PK sampling | PD sampling |
| --- | --- | --- | --- | --- | --- | --- |
| 1 | BE | HVs | 118 | 150 mg single dose,  in fasted or fed state | Full PK, 0-  48 h | 0,2,8,12h |
| 2 | Real-world  study | Patients | 167 | 110 mg twice dose,  in fasted or fed state | None | 2,12h |
