# Supplementary material for: Population pharmacokinetic analysis for dabigatran etexilate in Chinese patients with non-valvular atrial fibrillation
Source: Front Cardiovasc Med. 2022 Oct 28;9:998751. doi: 10.3389/fcvm.2022.998751 (PMC9650305; doi:10.3389/fcvm.2022.998751)
Supplement: Supplementary Table 2 — List of CYP3A4 and P-gps potent inducers and inhibitors. [file Table_2.docx]

Table S2. List of CYP3A4 and P-gps potent inducers and inhibitors

| No | Inducers | Inhibitors |
| --- | --- | --- |
| 1 | rifampicin | ketoconazole |
| 2 | phenytoin | itraconazole |
| 3 | [phenobarbital](javascript:;) | voriconazole |
| 4 | carbamazepine | posaconazole |
| 5 | St John's Wort | / |
