# Supplementary material for: Population pharmacokinetic analysis for dabigatran etexilate in Chinese patients with non-valvular atrial fibrillation
Source: Front Cardiovasc Med. 2022 Oct 28;9:998751. doi: 10.3389/fcvm.2022.998751 (PMC9650305; doi:10.3389/fcvm.2022.998751)
Supplement: Supplementary Table 3 — Population pharmacokinetic modeling process. [file Table_3.docx]

Table S3. Population pharmacokinetic modeling process

| No. | Model description | OFV | ΔOFV |
| --- | --- | --- | --- |
| Model including |  | | (df=1,p=0.05,3.84) |
| 1 | 1 compartment base model | 12705.611 |  |
| 2 | Add HDL on CL/F in model 1 | 12695.570 | -10.041 |
| 3 | Add AGE on CL/F in model 1 | 12697.658 | -7.953 |
| 4 | Add AGE on CL/F in model 2 | 12687.493 | -8.077 |
| Model excluding |  | | (df=1,p=0.001,6.63) |
| 5 | Remove AGE on CL/F in model 4 | 12695.570 | 8.077 |
| 6 | Remove HDL on CL/F in model 4 | 12697.658 | 10.165 |
